# Letter from Dr. Read, Correcting His Account of a Case of Superfœtation in a Mare, Published September, 1848

**Published:** 1848-11

**Authors:** Albert N. Read

**Affiliations:** Andover, Ashtabula Co., Ohio


### *Letter from* Dr. Read, *correcting his account of a Case of Superfœtation in a Mare, published September*, 1848.

Professor Huston:

My Dear Sir,—The time which elapsed between the two copulations in the case of Super-fœtation, published in the September number of the Examiner, is incorrect. It was only a little more than two days, (instead of two or three weeks.) I supposed the first statement was correct, but on visiting the township since, to learn the exact time, I find I was misinformed. I hasten to correct the statement, that I may not add another to the "*medical facts* that are *medical lies*." Yours truly,

Albert N. Read.

*Andover, Ashtabula Co., Ohio, Oct.* 1*st*, 1848.

